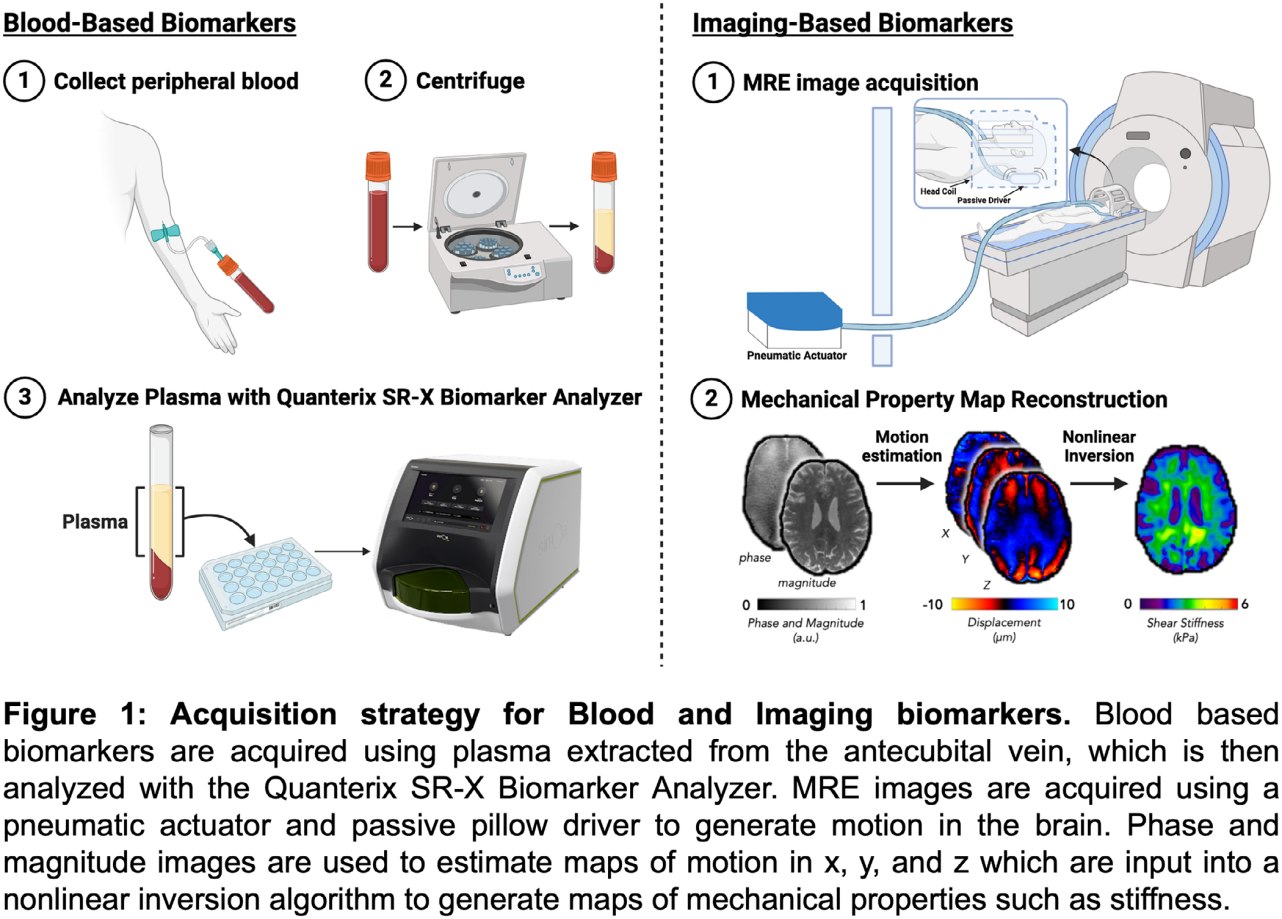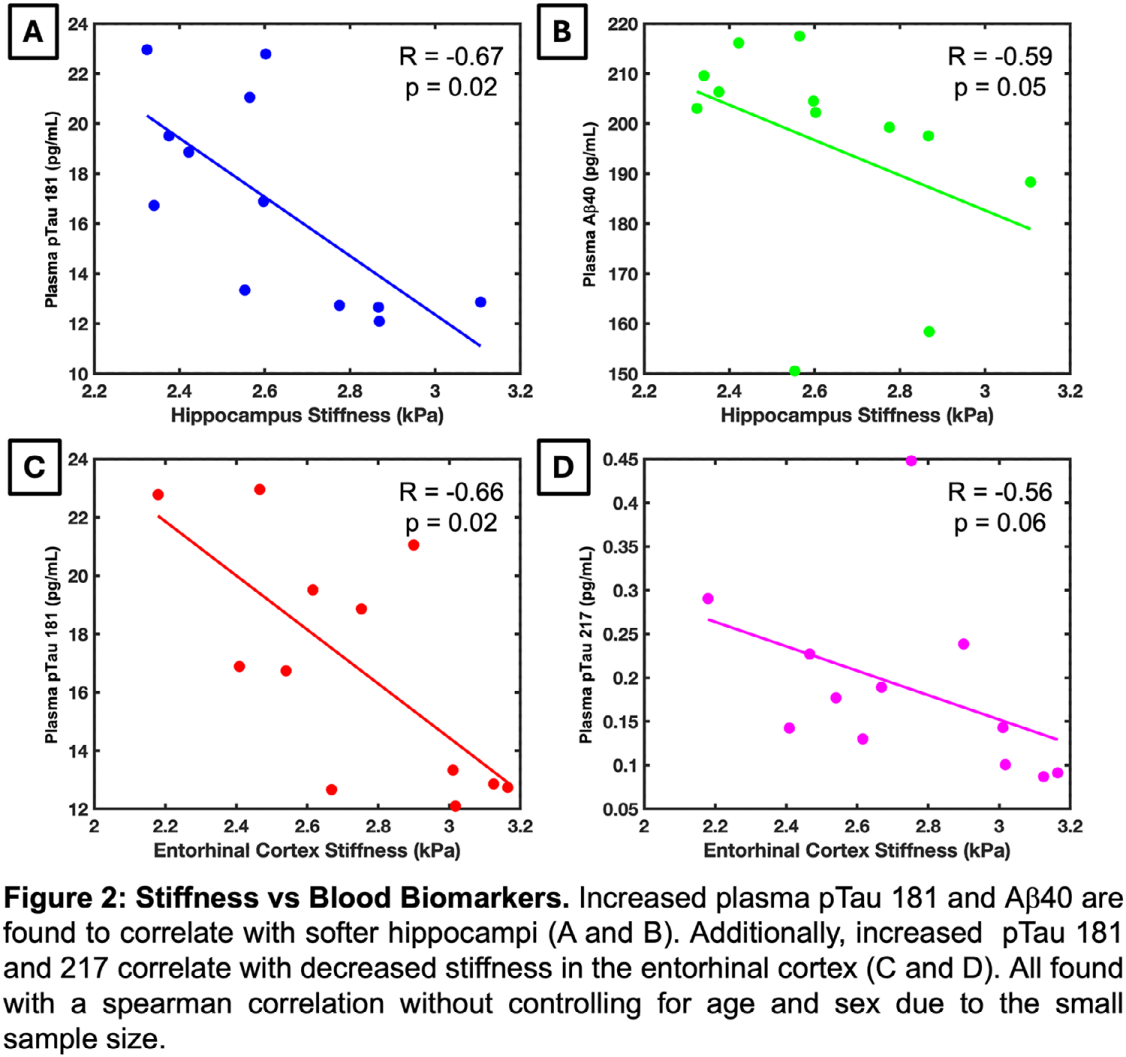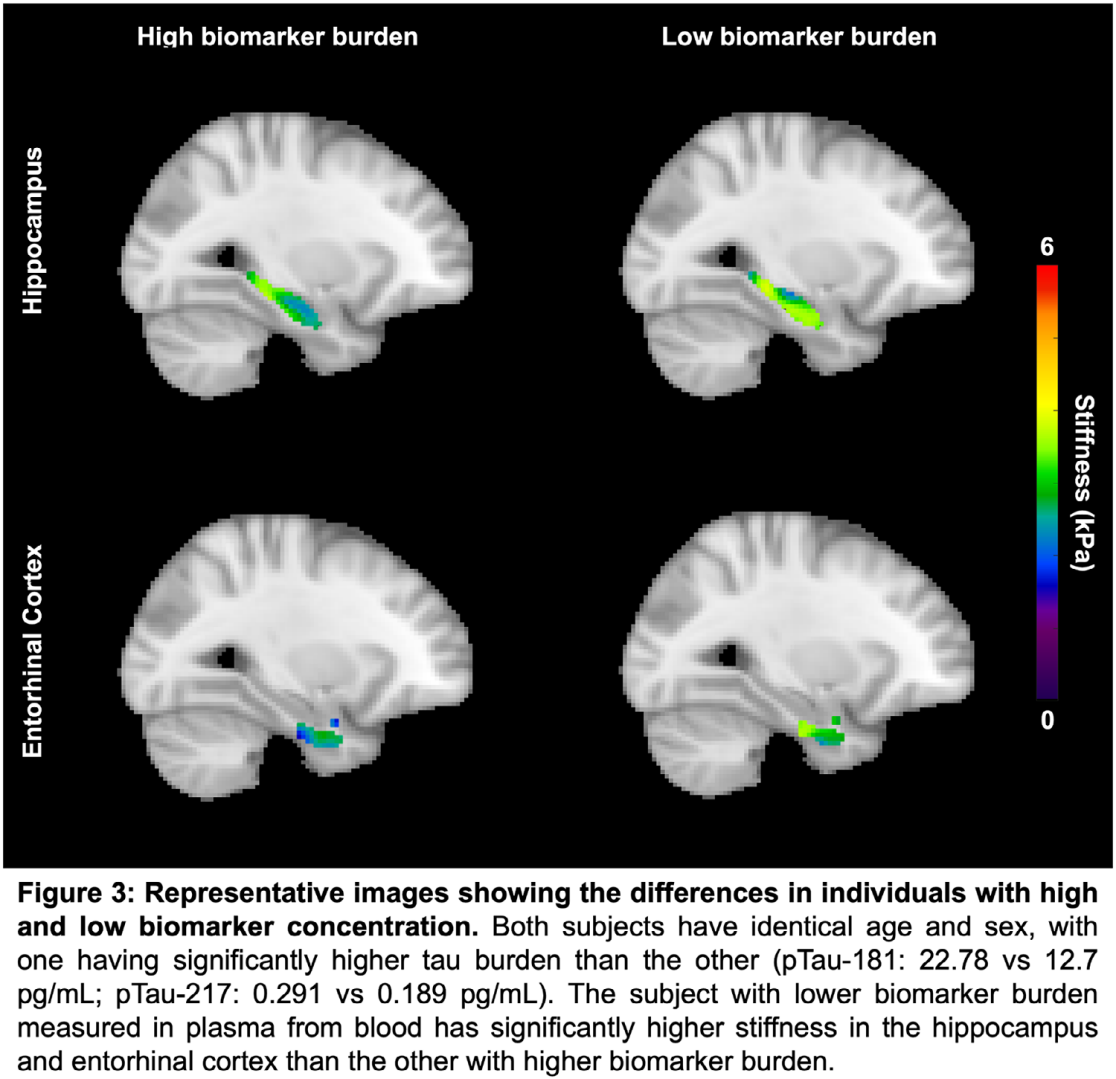# Increased Plasma Amyloid and Tau Concentrations associated with Decreased Mechanical Integrity of Braak Regions Measured with Magnetic Resonance Elastography

**DOI:** 10.1002/alz70856_101258

**Published:** 2025-12-25

**Authors:** Mary K Kramer, Nicholas J Pyontek, Nicholas A Rizzi, Kevin P Decker, Matthew L Cohen, Alyssa M Lanzi, Christopher R Martens, Curtis L Johnson

**Affiliations:** ^1^ University of Delaware, Newark, DE, USA

## Abstract

**Background:**

Up to two‐thirds of adults with Alzheimer's Disease (AD) biological pathology, including amyloid and tau accumulations, may remain asymptomatic. This asymptomatic stage of pathology may represent a crucial window for therapeutic intervention before the onset of clinical symptoms. Magnetic Resonance Elastography (MRE) is an MRI technique which uses applied mechanical vibrations to quantify brain tissue stiffness and has been used to quantify early AD‐related microstructural changes, particularly in the hippocampus and other regions known for early amyloid and tau accumulation.

**Method:**

Twelve individuals (46–81 years, 3M/9F) from the Delaware Longitudinal Study for Alzheimer's Prevention were included. These adults completed a robust neuropsychological battery, bloodwork, and imaging to determine elevated AD risk. Plasma biomarkers amyloid‐beta (Aβ) and phosphorylated tau (pTau‐181 and pTau‐217), were quantified using Single Molecule Array (Simoa) assays on the Quanterix SR‐X biomarker analyzer (Figure 1). MRE was then used to measure brain mechanics, specifically the stiffness of the hippocampus and entorhinal cortex (ERC), which have been identified as the first locations of amyloid and tau accumulation in Braak staging of AD.

**Result:**

Hippocampal stiffness showed inverse correlations with plasma biomarkers pTau‐181 (R=‐0.67, *p* = 0.02) and Aβ40 (R=‐0.59, *p* = 0.05). ERC stiffness exhibited a similar relationship with pTau‐181 (R=‐0.66, *p* = 0.02) and a near‐significant correlation with pTau‐217 (R=‐0.56, *p* = 0.06) (Figure 2). Representative images from age and sex matched individuals illustrate that individuals with lower AD biomarker concentrations have higher stiffness in the hippocampal and ERC regions, underscoring the link between AD plasma biomarker levels and brain tissue mechanical degradation (Figure 3).

**Conclusion:**

This work is the first to examine the relationship between the mechanical properties in the brain and plasma‐based amyloid and tau biomarkers. Reduced stiffness in the hippocampus and ERC likely reflect microstructural disruption from protein accumulation. These results in a small pilot cohort provide evidence supporting that MRE measures are sensitive to the impact on brain mechanics of these protein aggregates – measured with cost‐efficient and accessible blood‐based biomarkers for application in early detection of AD progression. Longitudinal data from this cohort will further clarify the dynamics of amyloid and tau accumulation and their impact on brain mechanics and cognitive decline.